# Reply to: Acute kidney injury and intra-abdominal hypertension in burn patients in intensive care

**DOI:** 10.5935/0103-507X.20190044

**Published:** 2019

**Authors:** Thalita Bento Talizin, Meiry Sayuri Tsuda, Marcos Toshiyuki Tanita, Ivanil Aparecida Moro Kauss, Josiane Festti, Cláudia Maria Dantas de Maio Carrilho, Cintia Magalhães Carvalho Grion, Lucienne Tibery Queiroz Cardoso

**Affiliations:** 1 Universidade Estadual de Londrina - Londrina (PR), Brasil.

The authors would like to express thanks for the comments and the opportunity to clarify the study points published in the Revista Brasileira de Terapia Intensiva^(1)^ about the incorporation of intra-abdominal pressure (IAP) monitoring in clinical practice.

Regarding the investigation of the use of glycopeptides, the authors clarify that this variable was treated as a potential confounding factor for the outcome "acute renal injury", which was explored in the study. This class of antimicrobials is known to be nephrotoxic^(2,3)^ and is widely used in our critically ill adult patients. Thus, this variable was included in the univariate analysis model together with other potentially confounding variables for the same outcome.

Regarding the continuous measurement of IAP using the AbViser(tm) system, which was described in the methods section, the authors agree with the comments because, as written, this description may cause confusion. The AbViser(tm) AutoValve(tm) IAP monitoring device is sterile, noninvasive, and disposable. It is coupled to the urinary catheter interruptedly. With the AbViser(tm), a measurement is obtained in 1 to 3 minutes, and measurements can be performed frequently but not continuously.

Finally, we agree that further studies are needed on the best routine for monitoring IAP in intensive care units, in addition to studies on the most appropriate conduct regarding altered IAP values. Team awareness of the benefits of this measure is valid for guiding treatments that avoid increasing IAP^(4)^ while minimizing organ dysfunction in critically ill patients.

*Thalita Bento Talizin Universidade Estadual de Londrina - Londrina (PR), Brazil.* *Meiry Sayuri Tsuda Universidade Estadual de Londrina - Londrina (PR), Brazil.* *Marcos Toshiyuki Tanita Universidade Estadual de Londrina - Londrina (PR), Brazil.* *Ivanil Aparecida Moro Kauss Universidade Estadual de Londrina - Londrina (PR), Brazil.* *Josiane Festti Universidade Estadual de Londrina - Londrina (PR), Brazil.* *Cláudia Maria Dantas de Maio Carrilho Universidade Estadual de Londrina - Londrina (PR), Brazil.* *Cintia Magalhães Carvalho Grion Universidade Estadual de Londrina - Londrina (PR), Brazil.* *Lucienne Tibery Queiroz Cardoso Universidade Estadual de Londrina - Londrina (PR), Brazil.*
